# The evolving role of ciclosporin in the management of vernal keratoconjunctivitis

**DOI:** 10.3389/fopht.2025.1525868

**Published:** 2025-04-25

**Authors:** Mumta Kanda, Bita Manzouri

**Affiliations:** Queen’s Hospital, Barking Havering and Redbridge University Hospitals NHS Trust, Romford, United Kingdom

**Keywords:** ciclosporin, cyclosporine, keratoconjunctivitis, VKC, ophthalmology, eye, paediatric

## Abstract

In the spectrum of allergic eye disease, vernal keratoconjunctivitis (VKC) is classed as one of the most severe disease entities and can have profound effects on visual development as well as on the emotional and psychological well-being of afflicted children. The traditional mainstay of treatment for the condition, to control the ocular inflammation, has been steroids but the use of these drugs has not been without side effects. Ciclosporin offers an alternative to steroids, providing symptom relief and control of the ocular inflammation, whilst averting the problems associated with raised intraocular pressure, cataract formation and reactivation of herpes simplex keratitis, all recognised side effects of topical steroids. However, the journey to the development of a formulation of an unpreserved ciclosporin for use in human eyes has been a protracted one; the aim of this article is to outline this journey and the role of ciclosporin in the modern management of this debilitating disease.

## Highlights

VKC is a potentially sight-threatening condition in children and adolescents that can have severe implications on quality of life, education, and mental wellbeing. Treatment can be variable due to limited data in the literature on best practice. It is widely acknowledged that steroids are the most effective for initial relief of acute flares but should not be used long-term due to side effects like glaucoma and cataract. Ciclosporin is an effective steroid sparing agent that has been used for the treatment of VKC since it was first approved in 1983. Significant developments in ciclosporin manufacturing over the years have produced a range of topical formulations with improving ocular bioavailability and some of these, including Restasis and Ikervis, were used off-label for the treatment of VKC. The VEKTIS study was the pivotal licensing trial showing that Verkazia, a cationic emulsion of 0.1% ciclosporin A, has proven efficacy in the treatment for moderate and severe VKC and is now a mainstay internationally in the treatment ladder to prevent flares and reduce long-term steroid use. Topical ciclosporin development has come a long way from use in rabbits and canines in the 1980s to the multiple formulations with good safety and efficacy available today. The safety of topical ciclosporin is a triumph and now gives community providers as well as hospital providers the confidence to prescribe ciclosporin.

## Introduction

Vernal keratoconjunctivitis (VKC) is an allergic eye disease that primarily affects children and teenagers with an estimated prevalence of up to 15% worldwide (1, 2). The pathogenesis of the disease is poorly understood but is thought to involve an immunologically mediated hypersensitivity reaction to environmental antigens (3). Although VKC typically resolves by puberty, it can cause sight-threatening and permanent corneal damage in severe cases (1). Symptoms from VKC can affect a child’s quality of life, mental health, and performance in education (4), as well as family dynamics; therefore, prompt diagnosis and adequate treatment is crucial. The mainstay of initial treatment of an acute VKC flare-up is ocular steroids, but long-term steroid use for repeated flare-ups can cause pathology such as glaucoma and cataract formation (5). Steroid-sparing agents have been used to control disease and prevent flare-ups of the disease whilst steroids are tapered, and as long-term immunomodulators. Ocular ciclosporin has emerged as a fundamental steroid-sparing agent with good efficacy in VKC. In this review, we discuss the mechanism of action, history of ciclosporin use in ophthalmology, formulations available and in development, and use in VKC.

### Immunopathophysiology and clinical features of VKC

VKC is considered a type IV hypersensitivity reaction. Although poorly understood, Th2 cells, Th9 cells, and mast cells are thought to be involved through production of pro-inflammatory mediators including interleukin (IL)3, IL4, IL5, IL9, IL13, and IL31 (6, 7). VKC clinically is divided into tarsal, limbal, and mixed subtypes based on the location of disease (8). The tarsal form is characterized by papillae, ranging in size from one mm to several mm, reminiscent of giant cobblestones, in the upper tarsal conjunctiva. The limbal form of VKC is characterized by gelatinous infiltrates of immune cells (eosinophils, macrophages, lymphocytes, plasma cells, basophils) and conjunctival goblet cells, Trantas’ dots (white nodules composed of epithelial debris and eosinophils), punctate keratitis, and shield ulcers (9). The mixed subtype has features of both. The eyelid margins are not involved. Limbal VKC is the predominate form in central and southern African countries, while the palpebral form is most frequent in Europe and the Americas (6, 8). Seasonal exacerbations, typically in spring/summer, may occur but up to 25% patients may develop chronic perennial disease (7, 9). Symptoms of VKC include itching, photophobia, epiphora, white mucous discharge, foreign body sensation, and pain (10). Sight-threatening complications of VKC include shield ulcers, infectious keratitis, corneal opacity/scarring, and limbal stem cell deficiency.

### Mode of action of ciclosporin

**Figure 1:**
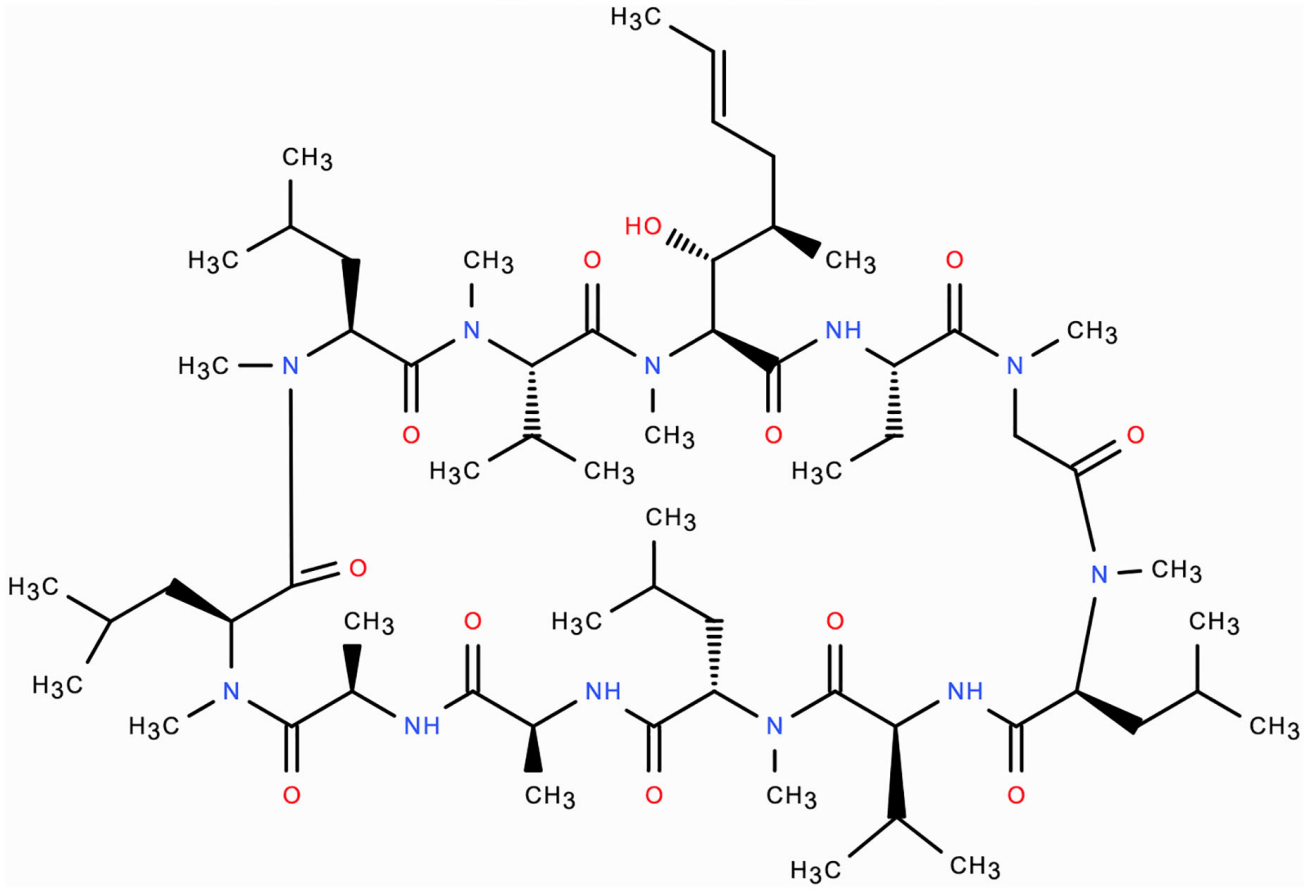
Molecular structure of ciclosporin: 11 amino-acid cyclic polypeptide 1202.6 Da in molecular weight.

Ciclosporin is a 1202.6 Dalton (Da) 11-amino acid cyclic polypeptide that is part of the calcineurin-inhibitor class of immunosuppressive drugs (**Figure 1**) (11). Ciclosporin enters T-cells and forms a protein complex with the cytoplasmic protein, cyclophilin A (12). This complex binds to and inhibits calcineurin, which is a calcium/calmodulin-dependent phosphatase enzyme. The inhibited calcineurin cannot dephosphorylate the nuclear factor of activated T-cells (NF-ATc) protein, which is subsequently unable to translocate into the nucleus and drive transcription of the IL2 gene. Therefore, ciclosporin ultimately functions by reducing IL2 formation by T-cells, which is needed for full T-cell activation (10, 11). It has also been postulated that ciclosporin induces T-cell apoptosis by regulating Fas/Fas ligand expression, caspase activation, and mitochondrial permeability transition pore (MPTP) opening (13). In contrast to its action on T-cells, ciclosporin may have a protective effect on conjunctival epithelial cells by inhibiting apoptosis, with significantly lower ocular side-effects than topical steroids (13).

### History of ciclosporin use and availability in ophthalmology

Ciclosporin was first isolated in 1970 from the soil fungus, *Tolypocladium inflatum*, by Jean Francois Borel at Sandoz Laboratories in Switzerland (14). Its potent immunosuppressive properties were highlighted when it successfully prevented renal allograft rejection when used in renal transplant recipients in 1978 (15). Ciclosporin (Sandimmune, Sandoz Pharmaceuticals, Basel Switzerland) was first approved by the Food and Drug Administration (FDA) in the United States (US) for immunosuppression in all organ transplant patients in 1983 (16). Subsequently, modified formulations of ciclosporin have been developed to improve its bioavailability for systemic use, such as Neoral (Novartis, Basel Switzerland) and Gengraf (AbbVie, North Chicago, Illinois USA), which are approved for several conditions such as rheumatoid arthritis, severe psoriasis, and severe atopic dermatitis (17). Over the 2021 calendar year, ciclosporin was the 175th most prescribed medication in the USA, with more than 2 million prescriptions (18).

In ophthalmology, ciclosporin was initially administered orally for the treatment of ophthalmic diseases, such as corneal graft rejection, uveitis, and VKC (17). Although oral ciclosporin was able to reach therapeutic levels in ocular tissues (19), the non-ocular administration led to the occurrence of systemic adverse events such as nephrotoxicity, hypertension, and anemia (20). Topical administration of ciclosporin (as 1% ciclosporin A in arachis oil) was investigated as early as 1981 to prevent corneal allograft rejection in rabbits (21). Canines were used to model use of topical 1% ciclosporin ointment for keratoconjunctivitis sicca in 1989 (22), which was soon followed by a pilot trial showing efficacy in humans in 1993 (23). In the years that followed, improvement of emulsion delivery systems that increased topical ciclosporin bioavailability led to the development of Restasis (ciclosporin A 0.05%, Allergan, Dublin Ireland). Restasis was used off-label for dry eye disease (DED) for several years, and eventually became the first topical ciclosporin approved by the FDA for DED in 2002 (24). Since then, further formulations have been developed and the use of ciclosporin has extended to it becoming a mainstay steroid-sparing agent in ocular inflammatory diseases such as uveitis and VKC, and in some cases for blepharokeratoconjunctivitis and long-term immunosuppression following corneal graft surgery.

### Development of topical ciclosporin formulations

**Figure 2:**
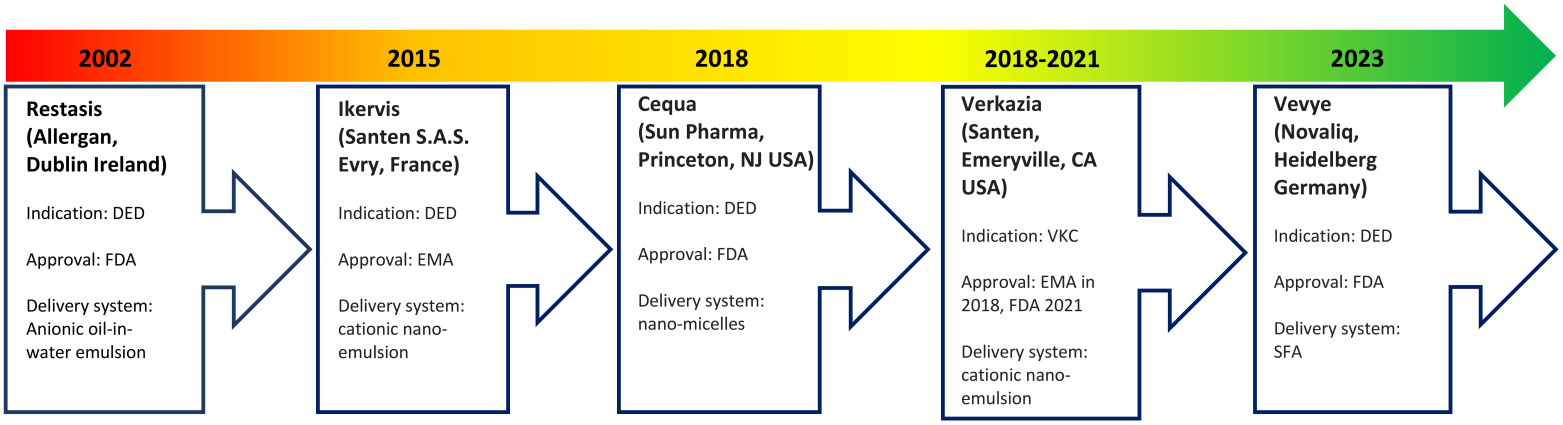
Timeline of development of topical ciclosporin formulations.

A continued challenge has been optimization of ocular tissue bioavailability of topical ciclosporin due to its large molecular weight, high hydrophobicity, and poor aqueous solubility (6, 16). Bioavailability is further reduced by static ocular barriers, such as the blood-aqueous barrier, and dynamic ocular barriers, such as tear turnover rate and tear dilution (25). As a result, several ciclosporin formulations with varying carrier delivery systems have been developed (**Figure 2**).

A lipophilic emulsion delivery system was the first to be approved by the FDA for DED in 2002. Restasis (Allergan, Dublin, Ireland) contains 0.05% ciclosporin A in a preservative-free anionic oil-in-water formulation comprising castor oil with polysorbate 80 and carbomer copolymer (17). However, release and intraocular penetration of ciclosporin from such emulsions is low, with time to onset of efficacy being six months on average (26). In addition, castor oil causes burning and pain, which reduces patient tolerance (27).

Restasis has been followed by several modified ciclosporin formulations. These include Ikervis (Santen S.A.S. Evry, France), which contains 0.1% ciclosporin in a cationic nano-emulsion. Ikervis was approved by the European Medicines Agency (EMA) in 2015 for DED (25). The cationic nano-emulsion was expected to have improved bioavailability and residence time compared with anionic emulsions due to the electrostatic interactions between the positively charged oil droplets and negatively charged mucins in the corneal epithelium (17).

Shortly after in 2018, the FDA approved Cequa (Sun Pharmaceutical Industries, Princeton, New Jersey USA), which contains 0.09% ciclosporin A in an aqueous nano-micellar formulation, for DED (28). These nano-micelles contain a hydrophobic core (sequestering the ciclosporin) surrounded by a hydrophilic shell, which may favour better dispersion and solubility of ciclosporin into the precorneal tear film (25).

In 2023 came the FDA approval of Vevye (Novaliq, Heidelberg Germany), which contains 0.1% ciclosporin A in a water-free solvent containing semi-fluorinated alkanes (SFA) (29). Liquid SFAs are amphiphilic compounds with low surface tension and low viscosity, which allows for a smaller drop size that spreads rapidly over the ocular surface to minimize reflex blinking and blurring of vision (30). This formulation was the first to show efficacy after four weeks of treatment (30). Further formulations unavailable in Europe and the USA exist in Asia and South America and more are in development (27).

A systematic review and meta-analysis comparing seven topical ciclosporins has shown that no single formulation was superior for all outcomes of DED treatment (27). Choice of ciclosporin will depend on local drug licensing, availability, cost, and presence of preservatives. Restatis, Cequa and Vevye are all preservative-free, but neither of these are approved for use in the EU. Whilst Ikervis contains ammonium cetalkonium chloride, this appears to neither have a preservative role or to cause corneal toxicity and has a similar safety profile to Restasis (31). Verkazia is the formulation of Ikervis that is approved for use in children and is chemically equivalent to it. In the UK, where a preservative-free topical ciclosporin ointment is required, Optimmune (Merck Animal Health, Rahway, New Jersey USA), which is a 0.2% ciclosporin ointment licensed for use in dogs but unlicensed in humans, is typically prescribed.

### Use of ciclosporin in VKC

The pathology of VKC is poorly understood but is thought to be a hypersensitivity reaction involving T-helper (Th) 2 cells, eosinophils, dendritic cells, and mast cells, as well as a variety of chemokines and cytokines (32). Therefore, a range of therapeutic approaches including antihistamines, mast cell stabilizers, nonsteroidal anti-inflammatory drugs, topical/injected (supratarsal)/oral steroids, immunomodulators, and targeted immunotherapeutic agents have been employed for the treatment of VKC (32). Topical steroids are widely acknowledged as the most effective first-line treatment for an acute flare of VKC, but a key limitation to their use is the risk of glaucoma and cataract development with long-term use, especially in those subset of patients who are children (33).

Ciclosporin (Ikervis and Restasis) has been used off-label as a steroid-sparing agent for VKC since it was first approved in 1983 (32). Topical 0.1% ciclosporin in a cationic emulsion, Verkazia (Santen, Emeryville, California USA), was approved by the EMA in 2018 and the FDA in 2021 (and subsequently several countries worldwide) for treatment of VKC in children 4 years and older and adolescents based on evidence presented in the pivotal VEKTIS trial. The VEKTIS study was a phase III, multi-center, double-masked, vehicle-controlled trial, in which patients aged 4-17 years with severe active VKC were randomized to one of three treatment arms over a four-month period. One arm received ciclosporin 0.1% cationic emulsion four times daily, the second arm received ciclosporin 0.1% twice daily with twice daily vehicle, and the third arm received vehicle alone four times daily (34). The study showed that 0.1% ciclosporin given twice or four times daily showed significant efficacy in reducing corneal inflammation and ocular itching as demonstrated by improved corneal fluorescein staining, reduced need for rescue medication, significantly improved patient symptoms and quality of life scores, and was well tolerated (34).

Verkazia became the first ciclosporin-based formulation approved specifically for VKC. Initially, its use was restricted to severe forms of the disease, as demonstrated in the pivotal VEKTIS study above, but more recent reports in the literature suggest that Verkazia may also be beneficial in moderate forms of VKC (defined as the presence of symptoms with photophobia but no corneal involvement) as well as in juvenile blepharokeratoconjunctivitis, expanding its potential clinical utilization (35). Emerging evidence indicates that Verkazia administered 2-4 times daily effectively alleviates symptoms and improves quality of life in patients with moderate VKC. This broader application could offer a valuable steroid-sparing option for patients experiencing persistent symptoms despite standard therapy (36). Verkazia has demonstrated a favourable safety profile in both clinical trials and post-marketing surveillance. The most common adverse events reported include mild ocular discomfort and transient blurred vision, which typically resolve without the need for treatment discontinuation. Unlike prolonged corticosteroid therapy, ciclosporin does not carry a significant risk of ocular hypertension or cataract formation (36).

However, there remains a considerable variation in the treatment of VKC, partly due to a paucity of data on effective treatments and best practice (4). In the UK, a review based on best practice across six large centres has provided some guidance on use of Verkazia in VKC (1). Treatment of VKC should be increased or decreased in a stepwise manner according to the severity of the disease. It is recommended that Verkazia is initiated as the topical steroids given for an acute flare-up are tapered in moderate and severe VKC. Severe VKC (defined as VKC with symptoms and corneal involvement, either superficial punctate keratopathy or corneal ulceration) may also benefit from systemic ciclosporin. The aim of ciclosporin treatment is to prevent further flare-ups and provide long-term disease control, but it can take a few weeks to show an effect. Adherence to treatment during this time is key and patients and their families should be made aware of the common side-effects of transient stinging and redness on drop instillation and the fact that these improve with long-term ciclosporin use, as this is a common cause of premature treatment cessation (1). The key to compliance is the appropriate counselling of patients to the use of Verkazia.

As part of the European Commission Pharmaceutical Strategy for Europe, the current recommended dose of Verkazia for children aged between 4-18 years is one drop four times a day (morning, noon, afternoon and evening), applied to each affected eye during the VKC season. If signs and symptoms of VKC persist after the end of the season, the treatment can be maintained at the recommended dose or decreased to one drop twice daily once adequate control of signs and symptoms is achieved. Treatment should be discontinued after signs and symptoms are resolved but can be reinitiated upon their recurrence. Efficacy and safety of Verkazia have not been studied beyond 12 months, nor is there evidence of use in children below four years of age (37).
